# Synthesis of Pd-Fe System Alloy Nanoparticles by Pulsed Plasma in Liquid

**DOI:** 10.3390/nano8121068

**Published:** 2018-12-18

**Authors:** Shota Tamura, Tsutomu Mashimo, Kenta Yamamoto, Zhazgul Kelgenbaeva, Weijan Ma, Xuesong Kang, Michio Koinuma, Hiroshi Isobe, Akira Yoshiasa

**Affiliations:** 1 Institute of Pulsed Power Science, Kumamoto University, Kumamoto 860-0862, Japan; 171d9101@st.kumamoto-u.ac.jp (S.T.); yamaken030806@gmail.com (K.Y.); jaza-86@mail.ru (Z.K.); 167d9102@st.kumamoto-u.ac.jp (W.M.); kangxuesong126@gmail.com (X.K.); 2 Faculty of Engineering, Kumamoto University, Kumamoto 860-0862, Japan; koinuma@chem.chem.kumamoto-u.ac.jp; 3 Faculty of Science, Kumamoto University, Kumamoto 860-0862, Japan; isobe@sci.kumamoto-u.ac.jp (H.I.); yoshiasa@sci.kumamoto-u.ac.jp (A.Y.)

**Keywords:** Pd-Fe alloy, nanoparticle, pulsed plasma in liquid

## Abstract

We synthesized Pd-Fe series nanoparticles in solid solution using pulsed plasma in liquid with Pd-Fe bulk mixture electrodes. The Pd-Fe atomic percent ratios were 1:3, 1:1, and 3:1, and the particle size was measured to be less than 10 nm by high-resolution transmission electron microscopy (HR-TEM). The nanoparticles showed face-centered cubic structure. The lattice parameter increased with increasing Pd content and followed Vegard’s law, and energy-dispersive X-ray spectra were consistent with the ratios of the starting samples, which showed a solid solution state. The solid solution structure and local structure were confirmed by HR-TEM and X-ray absorption fine structure.

## 1. Introduction

Pd is well-known as a hydrogen storage metal [1,2,3]. Pd-Fe alloy can be used to catalyze fuel cells and improve their conversion of chemical to electric energy. Furthermore, Pd-Fe alloy nanoparticles may increase the conversion rate of fuel cells. Research has shown that oxygen reduction of Pd-Fe catalyst is very stable in alkaline solution [4,5,6]. Mashimo’s laboratory has already synthesized Pt-Fe alloy nanoparticles [7]. Pd-Fe alloy nanoparticles in solid solution are expected to show excellent catalytic properties because of the interaction of 3d and 4d electrons. Pd-Fe alloy is half immiscible at room temperature.

Our previous studies have shown that pulsed plasma in liquid (PPL) is a good alternative method for synthesizing various nanomaterials [8]. This process is relatively cheap and environmentally friendly. The short duration (several microseconds) and quenching of the surrounding cool liquid limit the size of the crystals, which enables the synthesis of very small and/or metastable particles. We have synthesized nanoparticles of such materials as single elements [9], carbon-coated metals [10], fullerene [9], and onion-like carbon [11], and compound nanoparticles of oxides, carbides, and sulfides [12,13,14].

In this study, we seek to synthesize Pd-Fe alloy nanoparticles with atomic percent ratios of 1:3, 1:1, and 3:1 using PPL with Pd-Fe bulk mixture electrodes of the same composition. We expect that using melted Pd-Fe bulk electrodes will yield Pd-Fe solid solution nanoparticles because Pd and Fe ions exist in the environment of the pulsed plasma, so Pd-Fe clusters may easily form.

## 2. Materials and Methods

**Figure 1:**
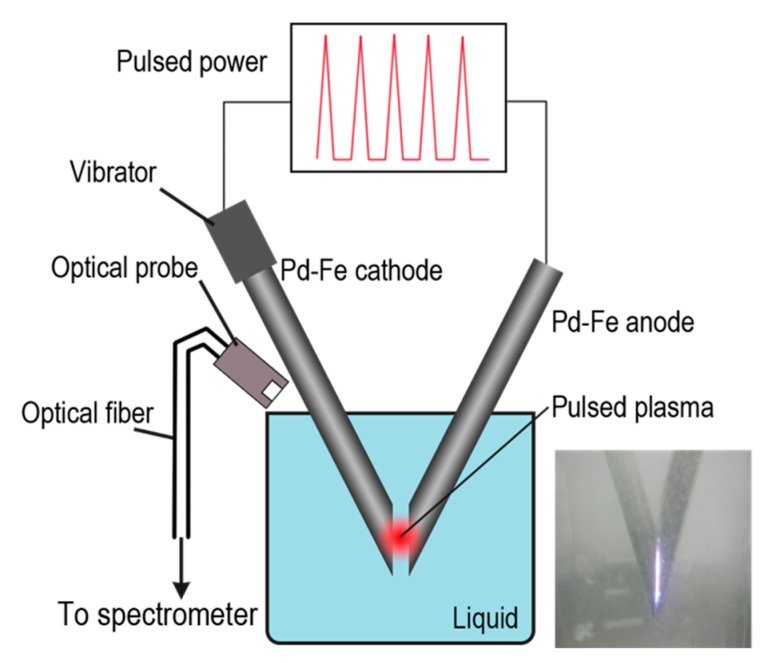
Schematic of pulsed-plasma-in-liquid method.

A schematic of the experimental setup is shown in Figure 1. Metal electrodes (cathodes and anodes) were submerged in a liquid connected to the power source. The impulse plasma was produced by the spark discharge between two electrodes. The gap between the two electrodes was approximately 0.2 mm, and the electrical current pulses that produced the pulsed plasma had a duration of about 20 μs. One of the electrodes was kept vibrating, so that the discharging could proceed continuously.

**Figure 2:**
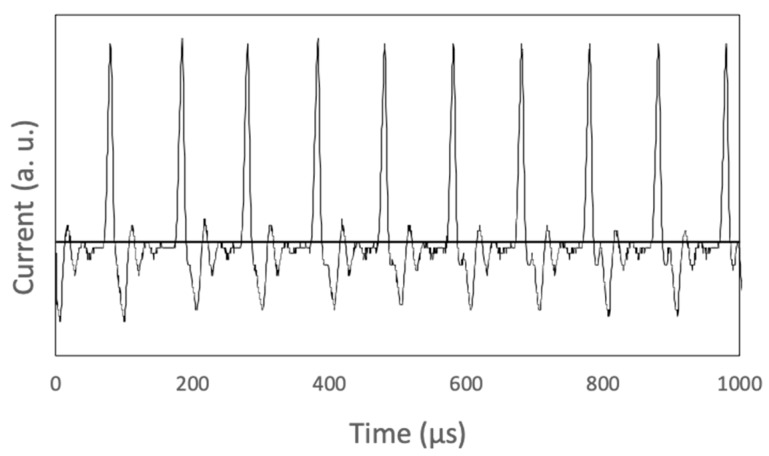
Waveform of output current.

In the plasma discharge experiment, we prepared two alloy bulk electrodes, each with a rod that was 4 mm in diameter and composed of pure Pd and Pd-Fe with atomic percent ratios of 1:3, 1:1, and 3:1. We first set the Pd-Fe electrodes into a 200-mL 99% ethanol solution and applied a pulsed voltage of 60.5 V for 60 min. Although the input voltage was set, the voltage applied to the electrodes varied with the discharge. Figure 2 shows the variation in output current. The current pulses were each about 10 μs with a rise time of less than 5 μs at intervals of about 100 μs. The current range was about 1.0–2.0 A. After the experiment, the samples were separated from the liquid into floating (upper) and sedimented (bottom) parts using a centrifuge, and then dried. Then we used an electric stove to dry these two parts for 4 h.

During the synthesis, atomic emission spectra of the plasma discharge were collected by an ALS SEC2000 UV-V optical spectrometer placed close to the plasma discharge zone outside the quartz beaker. Emission spectrum peaks were identified according to the NIST1 database [15]. 

The X-ray diffraction (XRD) patterns for the samples were measured with a Rigaku RINT-2500 VHF diffractometer, using CuKα radiation at 40 kV and 200 mA. We used high-resolution transmission electron microscopy (HR-TEM) (Philips Tecnai F20) to observe the morphology and microstructure of prepared samples. Elemental analysis was performed using a JEOL JSM-7600F energy-dispersive X-ray (EDX) spectroscope at 15 kV with a point resolution of 1.0 nm. We measured the X-ray absorption fine structure (XAFS) spectra near the Pd K-edge in transmission mode (with a beam size of 1.2 × 0.3 mm) at beamline NW10A AR, KEK, Tsukuba, Japan. Synchrotron radiation was monochromatized by a Si (311) double-crystal monochromater. X-ray energy calibration was performed by setting the copper metal pre-edge absorption peak to 8978.8 eV. Mirrors were used to eliminate higher harmonics. The radial structural function was obtained by performing a Fourier transform over the range 2.5 < *k* < 10.5 Å^−1^. We used an analytical edge XAFS (EXAFS) formula to carry out Fourier filtering and non-linear least-squares fitting of structural parameters. 

## 3. Results and Discussion

### 3.1. X-ray Diffraction

**Figure 3:**
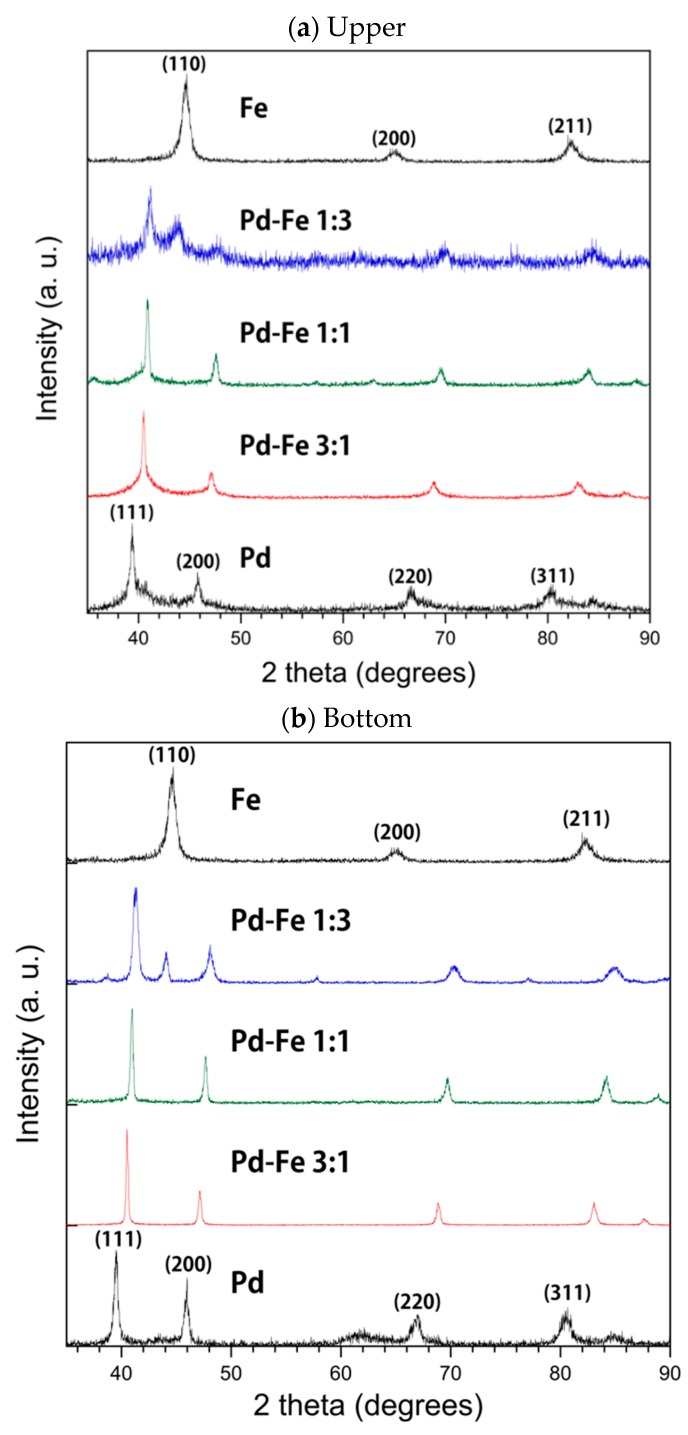
XRD patterns of Pd-Fe alloy nanoparticles and of elemental Pd and Fe nanoparticles in upper (**a**) and bottom (**b**) samples.

Figure 3 shows the XRD patterns of the floated (upper) (a) and sedimented (bottom) (b) samples of Pd-Fe alloy together with those of single-element Pd and Fe nanoparticles obtained by PPL. As the Pd concentration in samples increases, their XRD peaks become much more similar to those of Pd. Both the 1:1 and 3:1 Pd-Fe nanoparticles of the bottom and upper samples show the face-centered cubic (FCC) Pd phase in which all peaks are around 41 degrees, not the body-centered cubic (BCC) phase of Fe, which almost comes out at 45 degrees, while the 1:3 Pd-Fe shows both BCC and FCC phases. Each peak of the Pd phase shifts to larger 2θ, and those of the Fe phase shifts to smaller 2θ. As shown in Table 1, the lattice constants of the upper Pd-Fe alloy nanoparticles are slightly larger than those of the bottom ones. This may be caused by the larger surface area of the upper nanoparticles. The lattice parameter of the nanoparticles is smaller than that of pure Pd (3.9048 Å). This is because the ion radius of Pd is larger than that of Fe.

**Figure 4:**
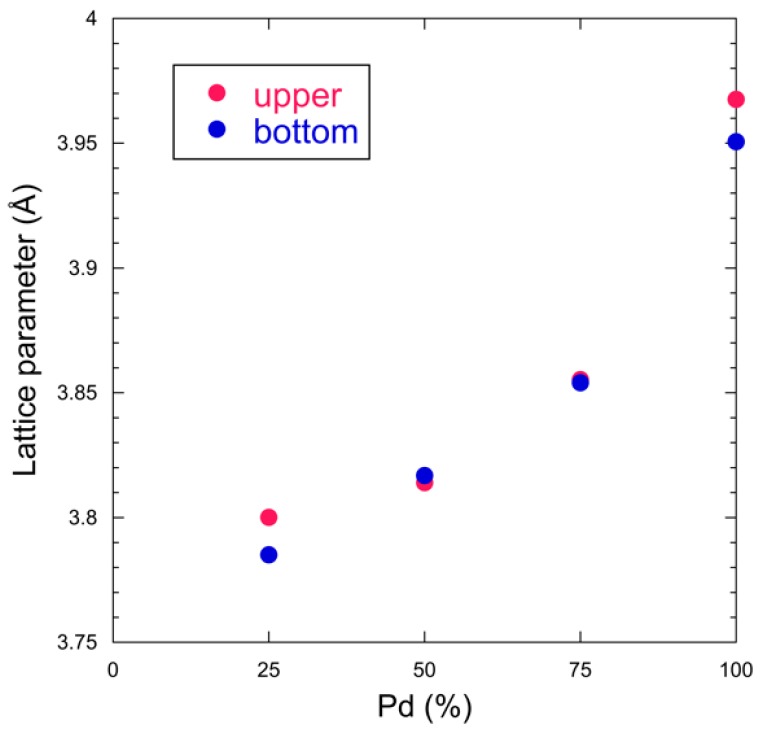
Lattice parameter versus Pd ratio of Pd-Fe alloy nanoparticles and pure Pd (100%) nanoparticles. The Pd-Fe nanoparticles have atomic percent ratios of 1:3, 1:1, and 3:1.

Figure 4 shows the lattice parameter versus composition. The lattice parameter increases with Pd ratio, which follows Vegard’s law. This shows that the synthesized Pd-Fe samples consist of solid solution nanoparticles with FCC structure.

We estimated the production rate of PdFe nanoparticles to be about 1.1 g per hour by measuring the weight of the electrodes. 

### 3.2. High-Resolution Transmission Electron Microscopy with Energy Dispersive X-ray Spectrometry

**Figure 5:**
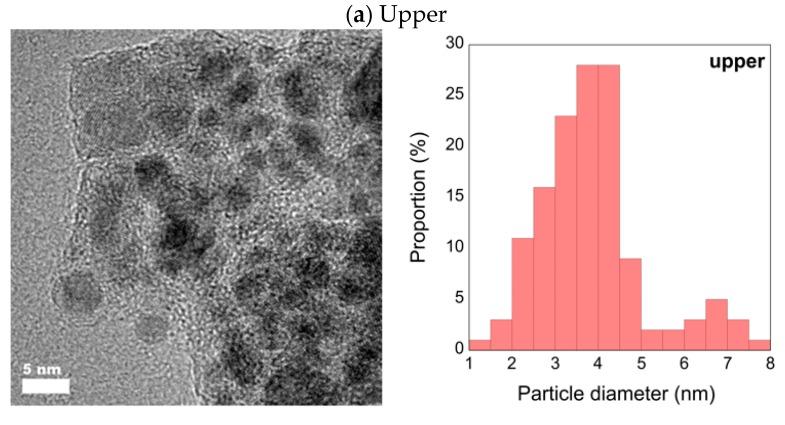

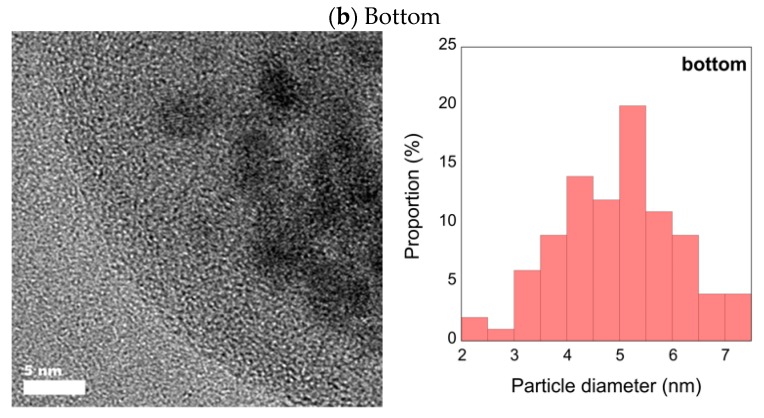
HR-TEM images of upper (**a**) and bottom (**b**) 1:1 Pd-Fe sample with particle diameter distribution.

**Figure 6:**
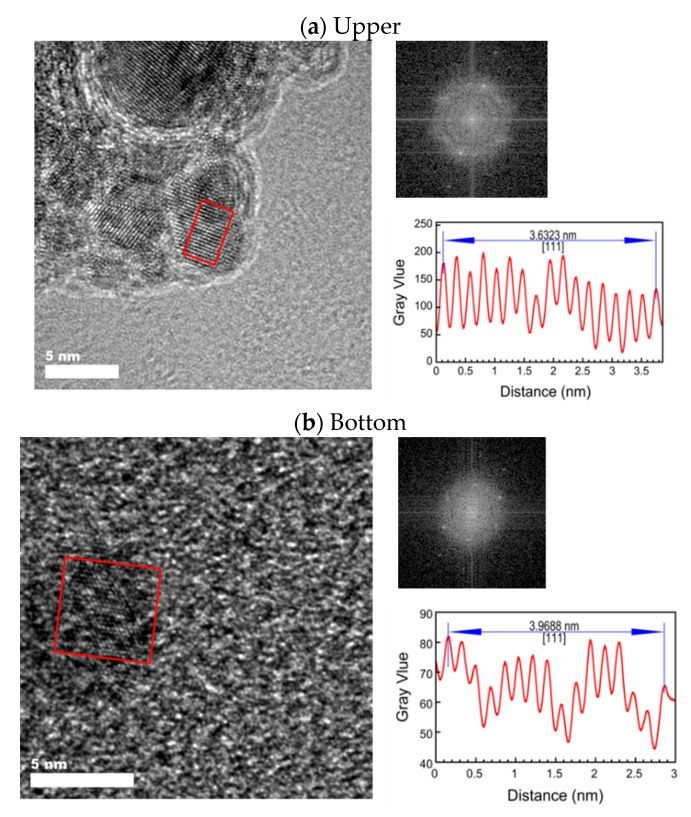
HR-TEM images of upper (**a**) and bottom (**b**) 1:1 Pd-Fe alloy nanoparticles with FFT pattern and surface spacing in the region enclosed in red.

Figure 5 shows the HR-TEM images of the upper and bottom samples of the 1:1 Pd-Fe alloy nanoparticles with the particle size distributions. We see from these figures that the average particle diameter of the upper is less than 4 nm and that of the bottom is less than 6 nm. Figure 6 shows the expanded HR-TEM images of selected areas and each corresponding fast Fourier transform (FFT) for the interplanar spacing of the 111 face, with average values of 2.270 Å (upper) and 2.089 Å (bottom). In this figure, we can see lattice ordering on the nano scale. 

**Figure 7:**
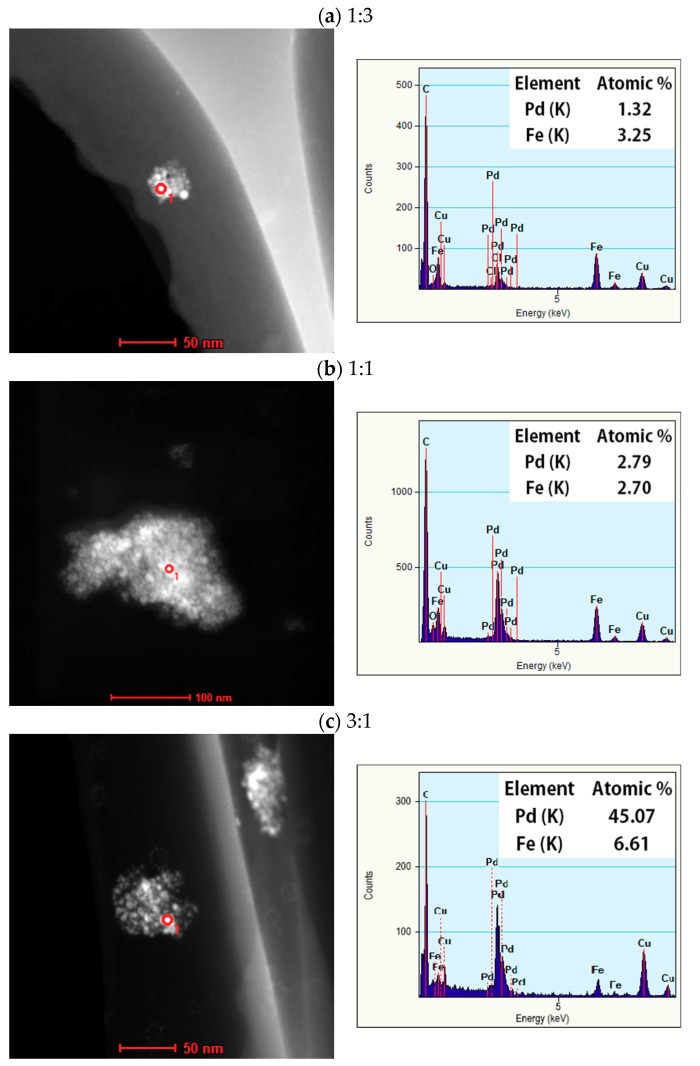
HAADF-STEM image and EDX analysis of upper 1:3 (**a**); 1:1 (**b**); and 3:1 (**c**) Pd-Fe alloy nanoparticles.

Figure 7a–c represents the HR-TEM EDX spectrum of the obtained nanoparticle when Pd:Fe = 1:3, 1:1, and 3:1, respectively. We measured the elemental composition at the point in each particle indicated by a red circle (<10 nm) in the high-angle annular dark-field scanning transmission electron microscopy (HAADF-STEM) image. The Pd-to-Fe atomic percent ratios for the 1:3, 1:1, and 3:1 samples were 1.3:3.2, 3.8:3.0, and 12.5:3.5, respectively, which are comparable to those of the starting bulk mixture electrode. 

From the XRD and HR-TEM results, we conclude that Pd-Fe solid solution nanoparticles with any composition ratios can be synthesized by PPL using Pd-Fe bulk mixture electrodes.

### 3.3. X-ray Absorption Near Edge Structure

**Figure 8:**
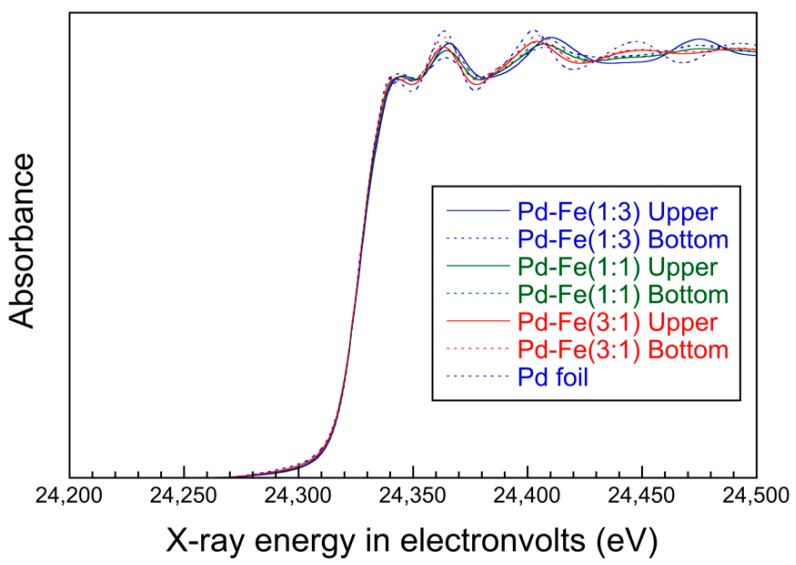
Normalized XANES of Pd parts of nanoparticles.

**Figure 9:**
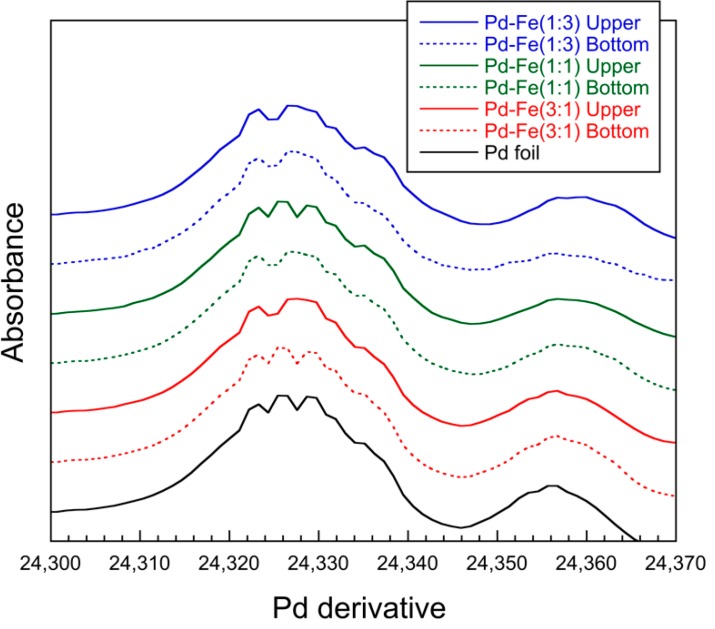
Derivative XANES of Pd parts of nanoparticles.

In Figure 8, the Pd K-edge X-ray absorption near-edge-structure (XANES) patterns of the synthesized nanoparticles are compared with those of the Pd foil. The Pd-Fe samples show a pattern similar to that of the Pd metal foil, though the amplitude of the sinusoidal structure decreases with increasing iron content. This indicates an increase in irregularity due to solid solution of different atomic sizes for iron. Figure 9 shows the first-derivative XANES spectra of the samples in Figure 8. The maximum peak in the first-derivative XANES spectrum in Figure 9, which corresponds to the energy with the largest slope in Figure 8, represents the threshold energy of the absorption edge. No chemical shifts of the threshold energies for Pd-Fe nanoparticles appear in Figure 8 or Figure 9. These results indicate that the samples keep the FCC structure and metallic character of Pd.

**Figure 10:**
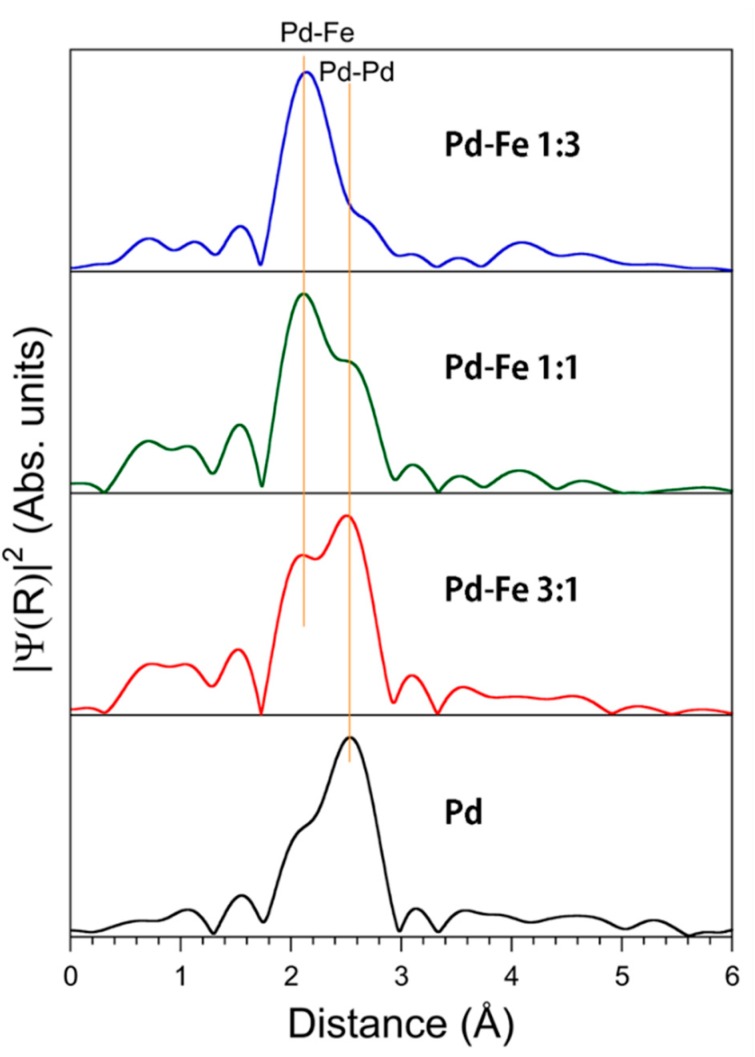
Fourier transforms of the Pd K-edge EXAFS oscillation function *k*^3^*χ*(k). No phase shift corrections are made. The first sets of nearest peaks represent the Pd-Fe and Pd-Pd distances.

From extended x-ray absorption fine structure (EXAFS) we obtained the radial distribution function and the interatomic distance from the X-ray absorbing atom to the adjacent atoms. Figure 10 shows the FFTs of the EXAFS oscillation function *k*^3^*χ*(k) showing the distances from the X-ray absorbing Pd atoms to the adjacent Pd and Fe atoms. The first and second peaks indicate the first set of nearest Pd-Fe and Pd-Pd distances, respectively. The shape of the Pd-Pd peak for pure Pd becomes asymmetric because of the backscattering amplitude. The Pd-Fe peak height increases and the Pd-Pd peak height decreases with increasing iron content.

Table 2 shows the local Pd-Pd and Pd-Fe interatomic distances determined from the EXAFS analyses. The Pd-Pd and Pd-Fe distances are 2.76 and 2.62 Å, respectively, indicating the distances for the coordination number of 12 in FCC structure. The Pd and Fe are mixed at the atomic level, which proves that the obtained sample is a Pd-Fe solid solution.

### 3.4. Atomic Emission Spectrum and Nanoparticle Formation Mechanism

**Figure 11:**
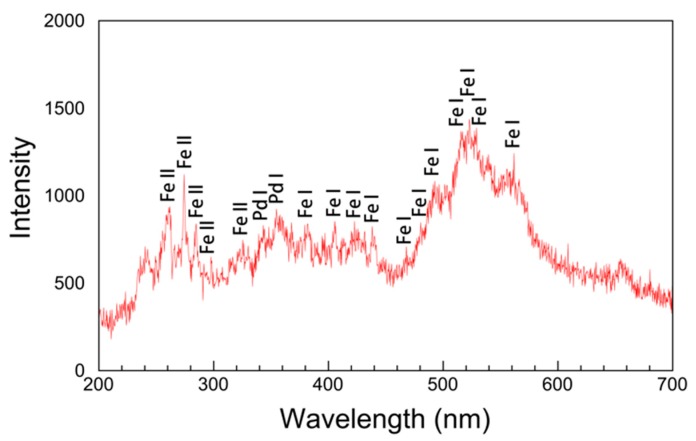
Optical emission spectrum from pulsed plasma in ethanol solution with Pd-Fe electrodes.

**Figure 12:**
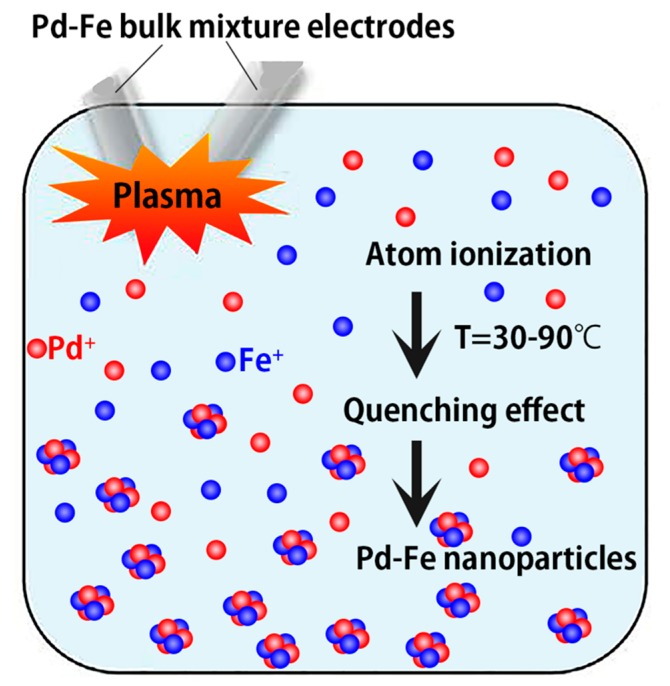
Illustration of Pd-Fe alloy nanoparticle formation via pulsed plasma in water.

Figure 11 shows the atomic optical emission spectra from the plasma discharge between the Pd-Fe electrodes submerged in ethanol. The main signals in the spectra belong to Fe (Fe^+^) and Pd (Pd^+^). The Pd lines are weak. The PdII lines may be too weak to be measured. The first and second ionization energies of Pd and Fe are 804.4, 1870 and 762.5, 1561.9 kJ/mol, respectively. We cannot observe the spectra of diatomic species such as OH, N2, CH, and C2. From the spectra of the Pd-Fe nanoparticles, we infer their formation mechanism via PPL as follows: Any raw materials, even those with high melting/boiling points, can be ionized by the high plasma temperature (2000–2500 °C). Therefore, Pd-Fe is ionized in the first stage, and positively charged Pd^+^ and Fe^+^ ions appear and disperse throughout the liquid. Then, Pd^+^ and Fe^+^ ions gather and form Pd-Fe nanoparticles under quenching by the surrounding cool liquid (Figure 12). The short pulse duration and rapid quenching prevent particle growth; therefore, the nanoparticles synthesized by PPL are very small. 

## 4. Conclusions

We have succeeded in synthesizing Pd-Fe series nanoparticles in solid solution with Pd:Fe composition ratios of 1:3, 1:1, and 3:1 by the PPL method using Pd-Fe alloy electrodes with the same compositions. The resulting 1:1 and 3:1 Pd-Fe particles are FCC while the 1:3 Pd-Fe particles are a mixture of BCC and FCC. The lattice parameter increases with increasing Pd concentration and follows Vegard’s law. The EDX and XAFS results also confirm the solid solution state. The sizes of the solid solution alloy particles are less than 10 nm. We expect synthesized Pd-Fe solid solution nanoparticles to show comparable or better catalytic properties than Pd nanoparticles.

## Figures and Tables

**Table 1:** Calculated lattice parameters of FCC phase of Pd-Fe alloy nanoparticles.

Lattice Parameter (Å)	Upper	Bottom
Pd	3.9676(39)	3.9506(37)
Pd_3_Fe	3.8553(102)	3.8540(1)
PdFe	3.8140(4)	3.8168(13)
PdFe_3_	3.8001(57)	3.7851(30)

**Table 2:** Local bonding distances (Å) in Pd-Fe alloy nanoparticles determined by EXAFS.

Sample	Pd-Pd Distance (Å)	Pd-Fe Distance (Å)
Pd Upper	2.76(1)	-
Pd_3_Fe Upper	2.76(1)	2.62(2)
PdFe Upper	2.74(2)	2.61(2)
PdFe_3_ Upper	2.75(2)	2.63(1)
